# Lung function benefits of traditional Chinese medicine Qiju granules against fine particulate air pollution exposure: a randomized controlled trial

**DOI:** 10.3389/fmed.2024.1370657

**Published:** 2024-04-29

**Authors:** Rucheng Chen, Lu Zhang, Weijia Gu, Ran Li, Huihua Hong, Linshui Zhou, Jinna Zhang, Yixuan Wang, Ping Ni, Shuqin Xu, Zhen Wang, Qinghua Sun, Cuiqing Liu, Junchao Yang

**Affiliations:** ^1^School of Public Health, Zhejiang Chinese Medical University, Hangzhou, China; ^2^Zhejiang International Science and Technology Cooperation Base of Air Pollution and Health, Hangzhou, China; ^3^First Affiliated Hospital of Zhejiang Chinese Medical University, Hangzhou, China

**Keywords:** fine particulate matter, lung function, traditional Chinese medicine, clinical trial, Qiju granules

## Abstract

**Introduction:**

Multiple targets are considered as the causes of ambient fine particulate matter [aerodynamic diameters of < 2.5 μm (PM_2.5_)] induced lung function injury. Qiju granules are derived from the traditional Chinese medicine (TCM) formula known as Qi-Ju-Di-Huang-Wan (Lycium, Chrysanthemum, and Rehmannia Formula, QJDHW), which has been traditionally used to treat symptoms such as cough with phlegm, dry mouth and throat, and liver heat. This treatment approach involves attenuating inflammation, oxidative stress, and fibrosis response. This study investigated the effects of Qiju granules on protecting lung function against PM_2.5_ exposure in a clinical trial.

**Methods:**

A randomized, double-blinded, and placebo-controlled trial was performed among 47 healthy college students in Hangzhou, Zhejiang Province in China. The participants were randomly assigned to the Qiju granules group or the control group based on gender. Clinical follow-ups were conducted once every 2 weeks during a total of 4 weeks of intervention. Real-time monitoring of PM_2.5_ concentrations in the individually exposed participants was carried out. Data on individual characteristics, heart rate (HR), blood pressure (BP), and lung function at baseline and during the follow-ups were collected. The effects of PM_2.5_ exposure on lung function were assessed within each group using linear mixed-effect models.

**Results:**

In total, 40 eligible participants completed the scheduled follow-ups. The average PM_2.5_ level was found to be 64.72 μg/m^3^ during the study period. A significant negative correlation of lung function with PM_2.5_ exposure concentrations was observed, and a 1-week lag effect was observed. Forced expiratory volume in one second (FEV_1_), peak expiratory flow (PEF), maximal mid-expiratory flow (MMEF), forced expiratory flow at 75% of forced vital capacity (FVC) (FEF_75_), forced expiratory flow at 50% of FVC (FEF_50_), and forced expiratory flow at 25% of FVC (FEF_25_) were significantly decreased due to PM_2.5_ exposure in the control group. Small airway function was impaired more seriously than large airway function when PM_2.5_ exposure concentrations were increased. In the Qiju granules group, the associations between lung function and PM_2.5_ exposure were much weaker, and no statistical significance was observed.

**Conclusion:**

The results of the study showed that PM_2.5_ exposure was associated with reduced lung function. Qiju granules could potentially be effective in protecting lung functions from the adverse effects of PM_2.5_ exposure.

**Clinical Trial Registration:**

identifier: ChiCTR1900021235.

## 1 Introduction

Ambient air pollution is an important global public health challenge. Epidemiological studies have demonstrated that exposure to particulate matter (PM_2.5_) increases mortality and morbidity in respiratory diseases (1, 2). The global burden of disease study estimated that 1.4 million deaths were due to respiratory diseases caused by ambient particulate matter alone (3), with PM_2.5_ identified as the most common detrimental factor among ambient particulate matter pollutants (3). The most effective way to minimize health hazards is by improving air quality by reducing pollutant release. Although more countries have been taking increased actions, over 90% of the people worldwide continue to breathe polluted air (4). Thus, personalized behavioral and preventive interventions may offer an alternative way to mitigate health hazards due to air pollution.

Previous studies have proved that both personal protective equipment and dietary supplementation could mitigate the adverse effects of PM_2.5_ air pollution (5–7). Health benefits have been demonstrated by using air purifiers and particulate-filtering respirators (5, 7). However, on many occasions, these interventions are not portable or readily available (e.g., outdoors and social interactions). Omega-3 fatty acid supplementation has been shown to alleviate some adverse effects in cardiovascular, metabolic, and neurological systems from air pollution (6). Given the role of the lung as the entrance of PM_2.5_ inhalational exposure, attenuating respiratory damage from air pollution should help prevent subsequential systemic injuries in our bodies. The exact pathophysiological mechanisms of lung injury are yet to be fully understood. Multiple targets including airway inflammation, oxidative stress, and pulmonary fibrosis are regarded as potential mechanisms that are involved in PM_2.5_-induced lung dysfunction (8). Therefore, it is reasonable to speculate that multi-target intervention of these biological pathways may offer more effective and preventive benefits against pulmonary injury from PM_2.5_ air pollution.

The traditional Chinese medicine (TCM) formula known as Qi-Ju-Di-Huang-Wan (Lycium, Chrysanthemum, and Rehmannia Formula, QJDHW) originates from “Yi Ji Juan Ba” of Dong Xiyuan of the Qing Dynasty (9). Qiju granules are derived from QJDHW, which has been traditionally used to treat symptoms such as cough with phlegm, dry mouth and throat, and liver heat (10, 11). In TCM's Five Phases Theory, Qiju granules are considered to have the properties of nourishing Yin and clearing heat with wood and fire attributes. Qiju granules contain five edible herbs, including *Rhodiola rosea L*. (Crassulaceae), *Portulaca oleracea L*. (Portulacaceae), *Lycium barbarum L*. (Solanaceae), *Chrysanthemum* × *morifolium (Ramat.) Hemsl*. (Asteraceae), and *Siraitia grosvenorii (Swingle) C.Jeffrey ex A.M.Lu & Zhi Y.Zhang* (Cucurbitaceae). These herbs are often used as a part of the regimen to treat lung disorders, which show attenuation in inflammation, oxidative stress, and fibrosis response (12–16). Our preliminary study has demonstrated the potential lung benefits of using Qiju granules against exposure to PM_2.5_ air pollution in an animal model (17). An oral administration of Qiju decoction decreases the concentration of tumor necrosis factor-α (TNF-α) and malondialdehyde (MDA), improving the expression of glutathione-peroxidase (GSH-PX) in serum (17). Qiju granules may act as a better alternative to attenuate lung injury from PM_2.5_. However, it remains unknown whether Qiju granules have protective effects against PM_2.5_-induced lung damage in humans.

In order to verify the effects of Qiju granules on humans, we conducted a randomized, double-blinded, and placebo-controlled trial to assess whether Qiju granules offered lung benefits against the adverse effects of PM_2.5_ exposure among healthy young adults.

## 2 Methods

### 2.1 Study participants and design

This trial was conducted from December 2018 to April 2019. We initially recruited 60 healthy college students at Zhejiang Chinese Medical University, Hangzhou, China, with the exclusion of students who are primary tobacco smokers or whose roommates are smokers, who are clinically diagnosed with cardiovascular or lung diseases, who had recent infection, who took antibiotics within the past month, or who had a history of allergies or family allergies. A total of 47 eligible participants were randomly assigned to two different treatment groups: the Qiju group and the placebo group. The randomization process was conducted using computer-generated random numbers. Gender was considered during randomization to ensure baseline comparability between the two groups. The random assignment procedure was carried out by independent researchers who were not involved in participant recruitment or diagnostic assessments, thus minimizing potential biases. All participants were intervened and monitored in a double-blinded manner throughout the study period. Two rounds of follow-up, with an interval of 2 weeks, were scheduled during a total intervention of 4 weeks. Data on individual characteristics (i.e., age, sex, height, and weight) and vital signs (i.e., heart rate (HR), blood pressure (BP), and respiration rate) were recorded, and lung function at the baseline and during the follow-ups was measured. Peripheral blood and the first-morning urine (midstream) samples were collected at the baseline and the end of the trial. Real-time PM_2.5_ exposure concentrations of those participants were also measured daily during the study time periods.

The study was registered in the Chinese Clinical Trial Registry (ChiCTR1900021235) on 13 February 2019 and was approved by the Ethics Committee of the First Affiliated Hospital of Zhejiang Chinese Medical University. All participants gave written informed consent at enrollment.

### 2.2 Ultra Performance Liquid Chromatography-Quadrupole-Time of Flight/Mass Spectrometry analysis of Qiju granules

The Ultra Performance Liquid Chromatography-Quadrupole-Time of Flight/Mass Spectrometry (UPLC-Q-TOF/MS) instrument was set to a capillary voltage of 2.5 kV (negative ion mode), sample cone voltage of 40 V, source temperature of 120°C, desolvation temperature of 500°C, and desolvation gas flow of 1000 L/h. Collision energy was set at 6 eV, and the full scan spectra were recorded from 50 to 1200 Da. Qiju granules were dissolved in purified water at a concentration of 100 mg/ml, and the solution was centrifuged to obtain the supernatant. The supernatant was analyzed by UPLC using reversed-phase C18 analytical columns (2.1 × 100 mm) packed with silica beads of 1.6 μm diameter (Cortecs^®^ UPLC^®^ T3, Waters, SYNAPT G2-Si, USA). The mobile phase consisted of 10% of solvent A (0.1% of formic acid) and 90% of solvent B (acetonitrile). The elution of Qiju granules was achieved over a period of 35 min with an injection volume of 2 μl and flow rates of 0.3 ml/min. The gradient elution program was set as follows: 0–2 min, 5% B; 2–32 min, 5–100% B; 32–33 min, 100% B; and 33–35 min, 5% B. The MS spectra of Qiju granules were acquired in negative-ion mode (Supplementary Figure S1).

### 2.3 Interventions

Qiju granules and placebo granules were provided by Huadong Medicine Co., LTD (Hangzhou, Zhejiang Province, China). Qiju granules were extracted and purified from *Rhodiola rosea L*. [Crassulaceae], *Portulaca oleracea L*. [Portulacaceae], *Lycium barbarum L*. [Solanaceae], *Chrysanthemum* × *morifolium (Ramat.) Hemsl*. [Asteraceae], and *Siraitia grosvenorii* (Swingle) *C.Jeffrey ex A.M.Lu & Zhi Y.Zhang* [Cucurbitaceae]. Placebo granules were purified from dextrin and caramel, with their appearance being identical and taste being similar to Qiju granules. The granules were stored at room temperature. Participants in the Qiju and control groups were instructed to dissolve 1 package of granules (15 g) in warm drinking water and take them orally every morning throughout the study period. The participants and researchers were blinded to either intervention.

### 2.4 Exposure assessment

To obtain real-time measurements of ambient PM_2.5_ concentrations, a portable environmental dust monitor (AirCasting Air Monitor Casing, New York Hall of Science, NY, USA) was provided to every two or three participants with similar activities who lived in the same dorm room. Enrolled participants were asked to wear the portable monitors from 8 am to 8 pm. The portable monitor was able to record ambient PM_2.5_ concentrations every second, and the data were collected to calculate daily average PM_2.5_ concentrations. To ensure the accuracy of the monitor, each portable monitor was calibrated through an aerosol monitoring meter (pDR-1500, Thermo Scientific, China) beforehand, and PM_2.5_ concentrations were calculated using the following formula:


$$\begin{array}{l} Formula\;1:\;Y=\frac{B_{\max}^{*}X}{K_{d}+X}, \end{array}$$

*Y* represents the concentrations of the portable monitor; *X* represents ambient PM_2.5_ concentrations; *B*_*max*_ represents the maximum specific binding in the same units as *Y*; and *K*_*d*_ represents the equilibrium dissociation constant, in the same units as *X*. The fitting degree of R^2^ was found to be between 0.90 and 0.98 in this formula (Supplementary Table S1, Supplementary Figure S2).

### 2.5 Health examination

At each round of health examinations, we measured the key characteristics of the participants, including height, body weight, blood pressure (BP), heart rate (HR), and lung function between 8:00 and 9:00 AM to minimize potential influences from circadian rhythms. Height and weight were measured by electronic scale without shoes and heavy coats, and then body mass index (BMI) was calculated. After the participant sat for at least 5 min, BP and HR were measured by an electronic sphygmomanometer (Omron J710, Japan). BP was measured using the left upper arm for at least three times at 1-min intervals. The mean values of the second and third HR, systolic BP (SBP), and diastolic BP (DBP) measurements were obtained. If the differences between the second and third readings of BP were >5 mmHg, additional measurements were obtained until satisfaction was achieved. Fasting peripheral blood and the first-morning midstream urine samples were collected between 8:00 and 9:00 AM. Routine blood and urine tests and hepatic and renal function examinations were conducted at the beginning and the end of the trial. A lung function detector (HI-101, Chestgraph, Tokyo, Japan) was utilized to measure the major parameters of lung function, including forced vital capacity (FVC), forced expiratory volume in one second (FEV_1_), peak expiratory flow (PEF), maximal mid-expiratory flow (MMEF), forced expiratory flow at 75% of FVC (FEF_75_), forced expiratory flow at 50% of FVC (FEF_50_), and forced expiratory flow at 25% of FVC (FEF_25_). Then, the ratio of the measured value to the predicted value was calculated as the lung function parameter.

### 2.6 Statistical analyses

PM_2.5_ concentrations and health data were associated with the time of lung function measured. Health data including lung function, BP, and HR were directly used as those were normally distributed. Linear mixed-effects models were applied to evaluate PM_2.5_ exposure concentrations in relation to each of the lung function parameters within each group. In the basic model, PM_2.5_ was included as a fixed-effect independent term, and a random-effect intercept was added for each subject to account for correlations among repeated measurements per person, assuming a compound symmetry covariance structure, which was further adjusted for sex and BMI. In order to explore the lag patterns regarding the impact of PM_2.5_ exposure on individual lung function, multiple averaging periods preceding the measurement of lung function were applied, i.e., lag 0 week (7 days before the examination day), lag 1 week (from 8 to 14 days before the examination day), and lag 0–1 week (14 days before the examination day). All linear mixed-effects models were conducted using the “lme4” package of R software version 3.3.1. In different group analyses, the difference in the lung function parameters and 95% CIs between the two groups were presented.

## 3 Results

### 3.1 Demographic characteristics

**Figure 1:**
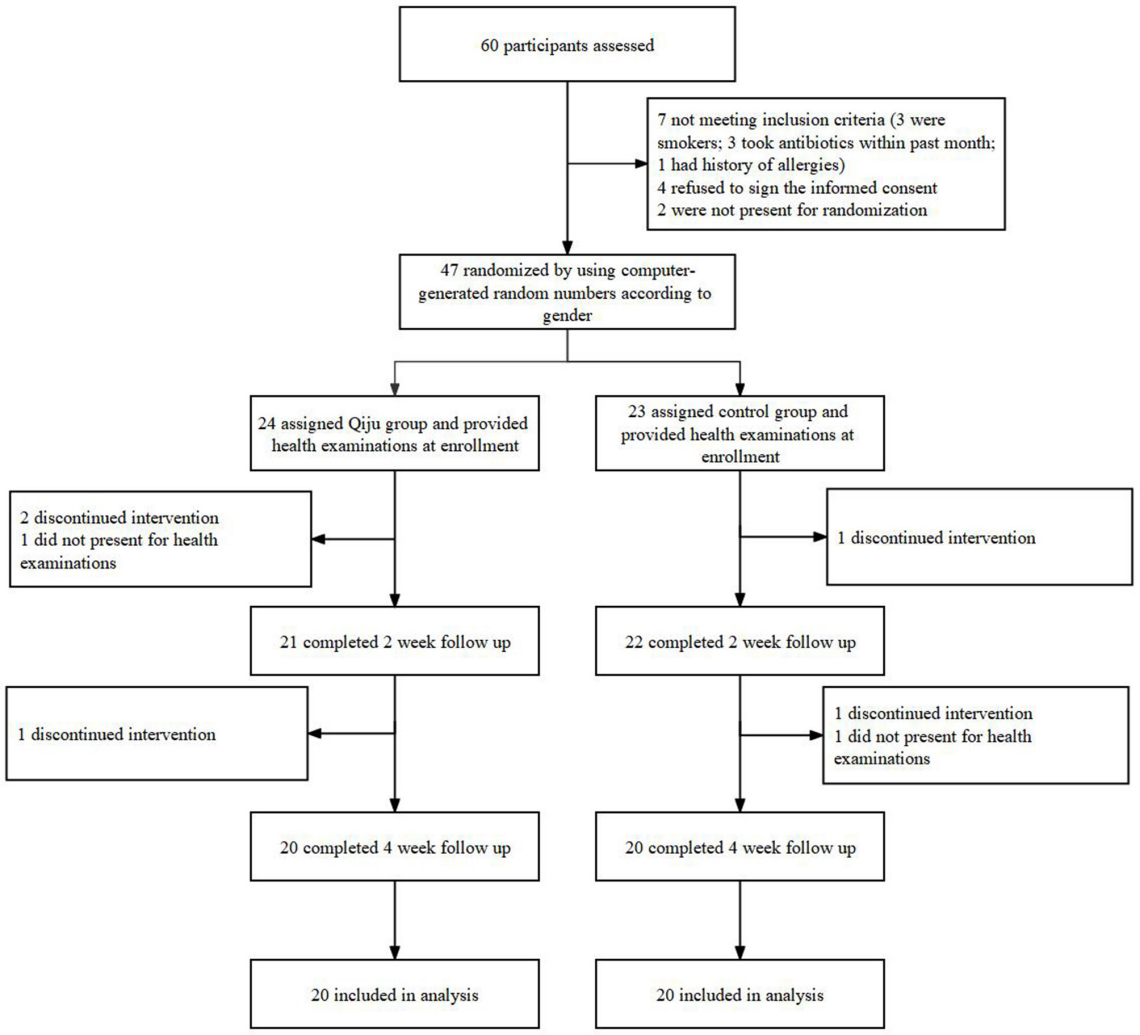
Flow diagram of enrollment and randomization. The intervention lasted for consecutive 4 weeks for both randomized arms. Of the 20 participants in the Qiju group who were included in the primary analysis, 3 participants discontinued intervention and 1 participant refused clinical follow-up. Of the 20 participants in the control group who were included in the primary analysis, 2 discontinued intervention and 1 refused clinical follow-up.

A total of 60 participants were screened, and 47 were enrolled and underwent randomization (24 were assigned to the Qiju group and 23 were assigned to the placebo group). Among those, 40 participants completed the scheduled follow-ups; of which 20 were in the Qiju group and 20 were in the control group (Figure 1). The participants in this study are aged between 19 and 22 years old, with 47.50% being male participants. There were no statistically significant differences in age or gender between the two groups. The other characteristics of these two groups were no significant differences at enrollment in the distributions of BMI, HR, SBP, DBP, respiratory rate (RPR), FVC, FEV1, PEF, MMEF, FEF75, FEF25, and FEF50 (Table 1). There was no statistically significant difference in biomarkers of the routine blood test, routine urine test, hepatic function, or renal function between these two groups at either baseline or at the end of the trial (Supplementary Table S2).

**Table 1:** Characteristics of the participants at baseline.

**Characteristics**	**Total population (*N =* 40)**	**Control group (*N =* 20)**	**Treatment group (*N =* 20)**	**t/chi value**	**P- value**
Sex				0.10	0.752
Male n (%)	19 (47.50)	9 (45.00)	10 (50.00)		
Female n (%)	21 (52.50)	11 (55.00)	10 (50.00)		
Age (year, Mean ± SD)	20.25 ± 0.954	20.05 ± 0.98	20.45 ± 1.00	1.34	0.188
BMI (Kg/m^2^, Mean ± SD)	20.87 ± 2.21	20.84 ± 2.04	20.89 ± 2.42	0.06	0.951
HR (/min, Mean ± SD)	70.23 ± 10.02	70.21 ± 11.34	70.25 ± 8.90	0.01	0.990
SBP (Mean ± SD)	110.72 ± 11.41	112.70 ± 13.77	108.75 ± 8.33	1.10	0.279
DBP (Mean ± SD)	75.03 ± 8.34	75.30 ± 9.91	74.75 ± 6.67	0.21	0.838
RPR (/min, Mean ± SD)	16.29 ± 2.74	15.94 ± 2.92	16.60 ± 2.60	0.39	0.701
FVC^*^ (%, Mean ± SD)	107.75 ± 12.99	109.11 ± 9.89	106.03 ± 15.60	0.75	0.460
FEV${\;}_{1}^{*}$ (%, Mean ± SD)	99.32 ± 7.59	98.67 ± 6.64	99.96 ± 8.55	0.53	0.597
PEF^*^ (%, Mean ± SD)	92.55 ± 18.75	94.02 ± 19.45	91.08 ± 18.47	0.47	0.645
MMEF^*^ (%, Mean ± SD)	97.67 ± 20.04	97.33 ± 19.41	98.00 ± 21.15	0.11	0.917
FEF${\;}_{75}^{*}$ (%, Mean ± SD)	84.74 ± 24.05	82.33 ± 20.20	87.15 ± 27.70	0.63	0.534
FEF${\;}_{50}^{*}$ (%, Mean ± SD)	93.98 ± 19.71	94.27 ± 19.48	93.69 ± 20.44	0.09	0.927
FEF${\;}_{25}^{*}$ (%, Mean ± SD)	95.35 ± 18.39	96.36 ± 20.00	94.28 ± 17.01	0.35	0.729

^*^Percentage of predicted value. BMI, body mass index; HR, heart rate; SBP, systolic blood pressure; DBP, diastolic blood pressure; RPR, respiration rate; FVC, forced vital capacity; FEV_1_, forced expiratory volume in one second; PEF, peak expiratory flow; MMEF, maximal mid-expiratory flow; FEF_75_, forced expiratory flow at 75% of FVC; FEF_50_, forced expiratory flow at 50% of FVC; FEF_25_, forced expiratory flow at 25% of FVC.

### 3.2 Monitoring of PM_2.5_ concentration

**Figure 2:**
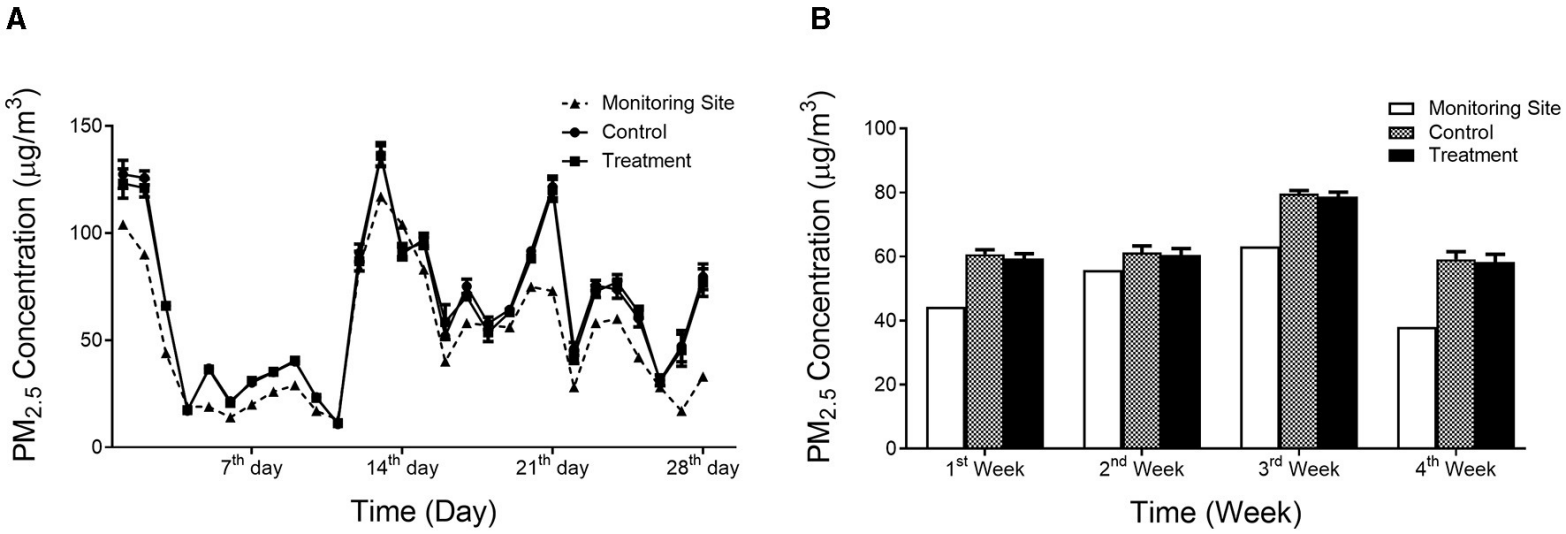
PM_2.5_ concentrations during the study period. **(A)** average daily PM_2.5_ concentrations; **(B)** average weekly PM_2.5_ concentrations. Values are expressed as mean ± SEM.

The average concentrations of individual PM_2.5_ exposure among participants were 64.72 ± 34.98 μg/m^3^ during the study period, which is slightly higher than that from the average level of Hangzhou city. At the individual level, daily and weekly averages of PM_2.5_ concentrations aligned well between the two groups during the study period (Figures 2A, B). The exposure of PM_2.5_ concentrations during different time periods before the conduction of the health examination was also calculated. The average concentrations at lag 0 week (7 days before the examination day), lag 1 week (from 8 to 14 days before the examination day), and lag 0–1 week (14 days before the examination day) were 57.58 ± 6.26 μg/m^3^, 80.12 ± 6.53 μg/m^3^, and 68.85 ± 4.63 μg/m^3^, respectively (Supplementary Table S3).

### 3.3 Correlation of lung function with PM_2.5_ concentrations

Lung function was primarily measured by FVC, FEV_1_, MMEF, PEF, FEF_25_, FEF_50_, and FEF_75_. The majority of these parameters were significantly negatively correlated with PM_2.5_ exposure concentrations at lag 1 week except FVC. None of these parameters was significantly correlated with PM_2.5_ exposure concentrations at lag 0 week. The majority of these parameters of lung function was not significantly correlated with PM_2.5_, and only PEF and PEF_25_ were significantly correlated with PM_2.5_ exposure concentrations at lag 0–1 week (Table 2).

**Table 2:** Correlation between lung function and PM_2.5_ exposure concentrations at different periods before health examination^#^.

**Lung function (%)**	**Lag 0 week^&^**	**Lag 1 week^&^**	**Lag 0–1 week^&^**
FVC	−0.07	−0.24	−0.12
FEV_1_	0.10	−0.51^*^	−0.10
MMEF	0.24	−0.60^*^	0.02
PEF	−0.15	−0.46^*^	−0.43^*^
FEF_25_	0.06	−0.50^*^	−0.33^*^
FEF_50_	0.24	−0.57^*^	0.02
FEF_75_	0.22	−0.45^*^	0.05

^#^r was calculated by correlation analysis at different periods before the health examination. ^*^significant of r value between lung function and PM_2.5_ concentrations, P < 0.05. ^&^Lag 0 week means 7 days before the examination day; lag 1 week means from 8 to 14 days before the examination day; lag 0–1 week means 14 days before the examination day. FVC, forced vital capacity; FEV_1_, forced expiratory volume in one second; PEF, peak expiratory flow; MMEF, maximal mid-expiratory flow; FEF_75_, forced expiratory flow at 75% of FVC; FEF_50_, forced expiratory flow at 50% of FVC; FEF_25_, forced expiratory flow at 25% of FVC.

### 3.4 Percentage changes in lung function

**Figure 3:**
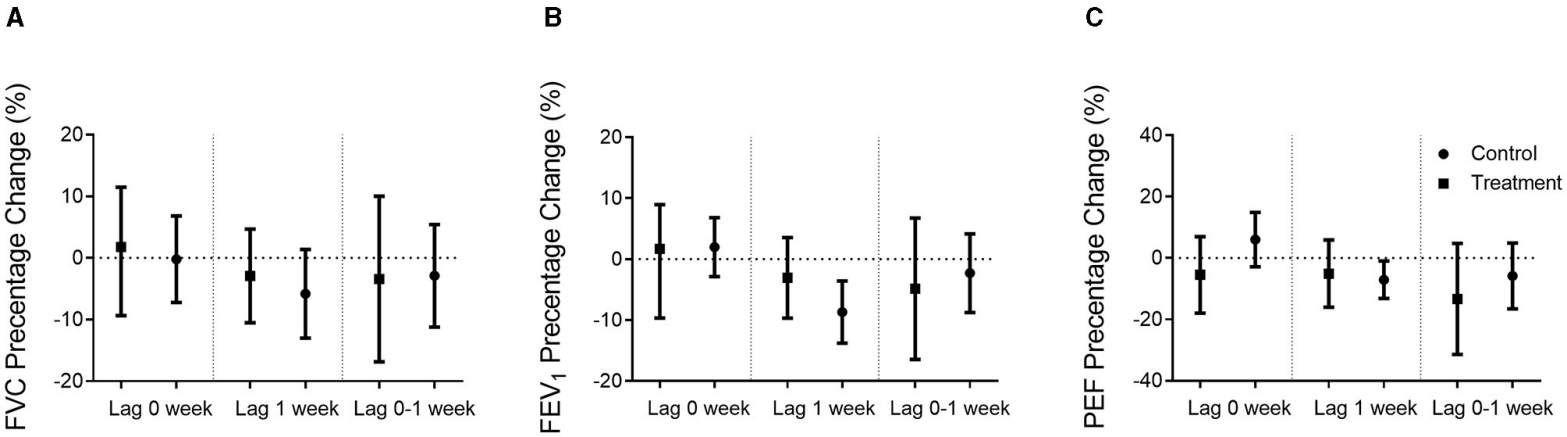
Percentage changes in airway function when adjusted sex and BMI. Percentage changes (mean and 95% confidence intervals) in airway function per 10 μg/m^3^ increase in PM_2.5_ exposure concentrations in the Qiju group and the control group at different lag times were shown. **(A)** FVC percentage of the predicted value change. **(B)** FEV_1_ percentage of the predicted value change. **(C)** PEF percentage of the predicted value change. Values are expressed as mean ± SEM. FVC, forced vital capacity; FEV_1_, forced expiratory volume in 1 s; PEF, peak expiratory flow.

**Figure 4:**
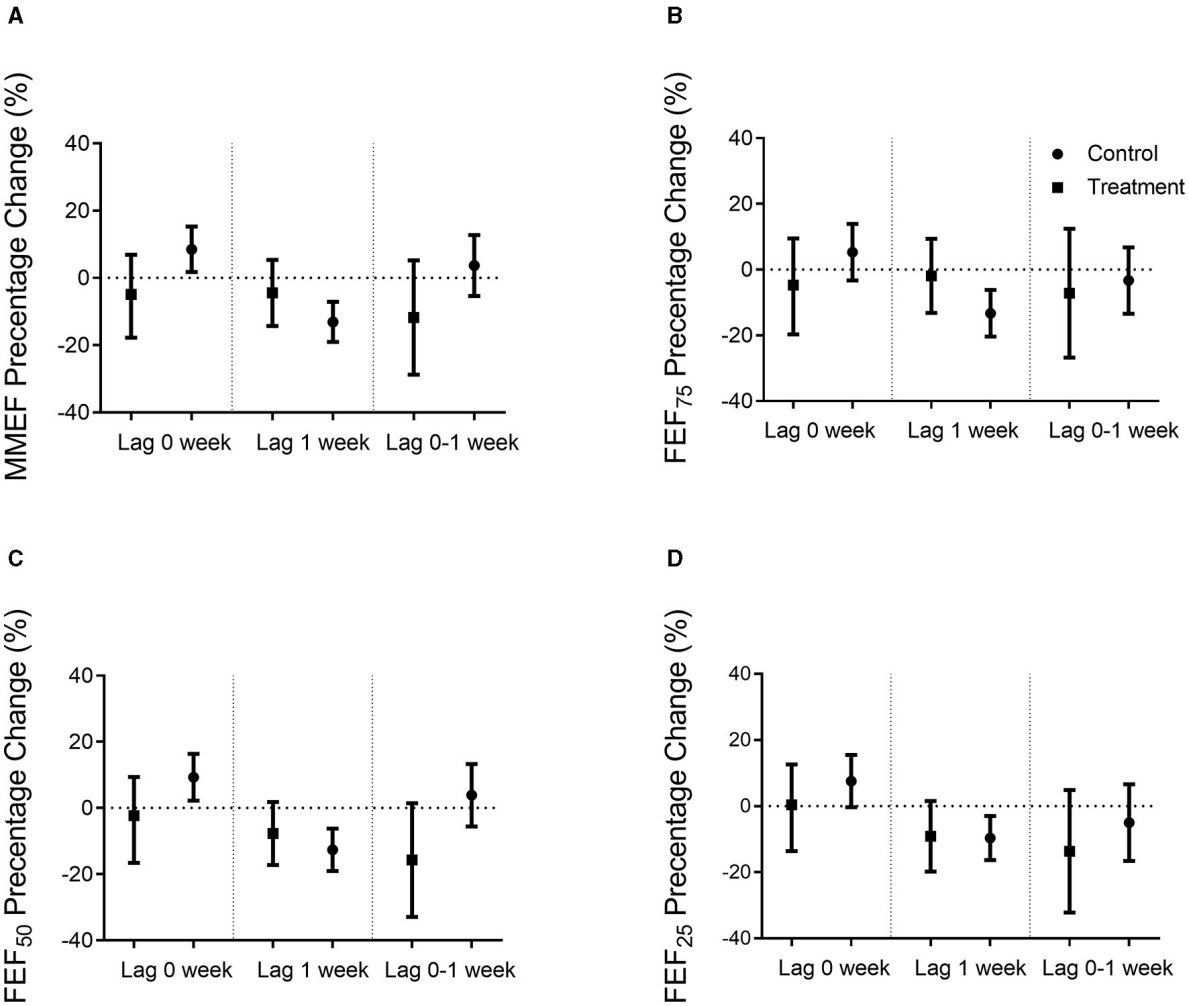
Percentage changes in small airway function when adjusted sex and BMI. Percentage changes (mean and 95% confidence intervals) in small airway function per 10 μg/m^3^ increase in PM_2.5_ exposure concentrations in the Qiju and the control groups at different lag times were shown. **(A)** MMEF percentage of the predicted value change. **(B)** FEF_75_ percentage of the predicted value change. **(C)** FEF_50_ percentage of the predicted value change. **(D)** FEF_25_ percentage of the predicted value change. Values are expressed as mean ± SEM. MMEF, maximal mid-expiratory flow; FEF_75_, forced expiratory flow at 75% of FVC; FEF_50_, forced expiratory flow at 50% of FVC; FEF_25_, forced expiratory flow at 25% of FVC.

Overall, the majority of the parameters related to lung function was significantly decreased in response to PM_2.5_ exposure concentration at lag 1 week in the control group. However, weaker associations with no statistical significance were observed in the Qiju group. Lung function did not vary significantly at lag 0 week and 0–1 week in response to PM_2.5_ exposures in either group (Figures 3, 4).

### 3.5 Percentage changes in large airway function

As shown in Figure 3, FEV_1_ and PEF, which are the major parameters of large airway function, were negatively correlated with PM_2.5_ exposure concentrations in the control group at lag 1 week, but not statistically significant in the Qiju group. In the control group, there was an 8.80% (95% CI: 3.54% to 13.74%) decrease in the percentage of estimate FEV_1_ and a 7.07% (95% CI: 1.00% to 13.21%) decrease in the percentage of estimate PEF per 10 μg/m^3^ increase in PM_2.5_ at lag 1 week. However, the changes in FEV_1_ and FEF were not significant in the Qiju group with only 2.86% (95% CI: −3.45% to 9.77%) and 4.98% (95% CI: −5.82% to 13.21%) decrease in the percentage of estimate FEV_1_ and FEF, respectively, at lag 1 week. The association between FVC and PM_2.5_ exposure concentrations was not significant at each time point in both groups (Figure 3).

### 3.6 Percentage changes in small airway function

As shown in Figure 4, MMEF, FEF_75_, FEF_50_, and FEF_25_, which are the major parameters of small airway function, were negatively correlated with PM_2.5_ exposure concentrations in the control group at lag 1 week, but not statistically significant in the Qiju group. In the control group, there was 13.07% (95% CI: 7.12% to 19.03%) decrease in the percentage of estimate MMEF, 13.38% (95% CI: 6.12% to 20.30%) decrease in the percentage of estimate FEF_75_, 12.53% (95% CI: 6.31% to 19.08%) decrease in the percentage of estimate FEF_50_, and 9.67% (95% CI: 2.95% to 16.26%) decrease in the percentage of estimate FEF_25_ per 10 μg/m^3^ increase in PM_2.5_ at lag 1 week. However, the changes in these parameters were not significant in the Qiju group with only 3.71% (95% CI: −4.98% to 14.63%), 4.98% (95% CI: −9.06% to 13.47%), 6.81% (95% CI: −1.36% to 17.68%), and 8.84% (95% CI: −1.46% to 19.92%) decrease in the percentage of estimate MMEF, FEF_75_, FEF_50_, and FEF_25_, respectively, at lag 1 week (Figure 4).

### 3.7 The difference in airway function between groups when PM_2.5_ was adjusted

**Figure 5:**
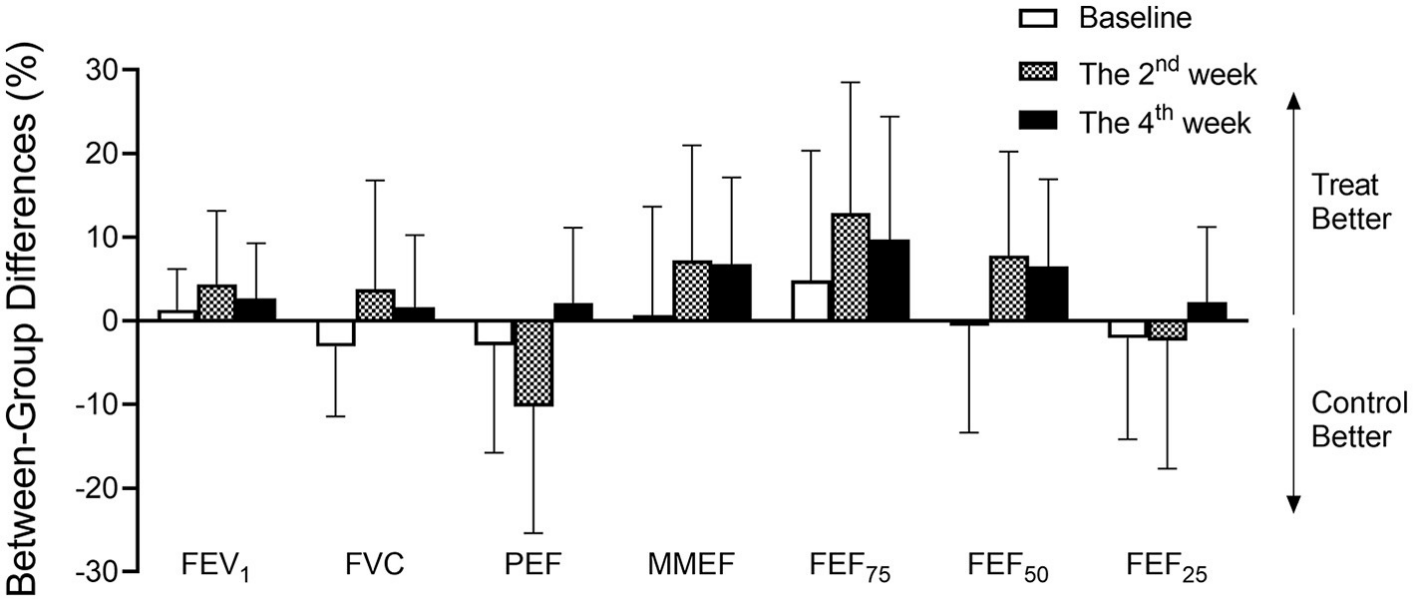
The difference in airway function between groups when adjusted PM_2.5_ concentrations. Different percentages (mean and 95% confidence intervals) in airway function between the Qiju group and the control group in different intervention times (baseline, the 2nd week, and the 4th week). PM_2.5_ exposure concentrations were adjusted in the 2nd and 4th weeks. Values are expressed as mean ± SEM. FVC, forced vital capacity; FEV_1_, forced expiratory volume in 1 s; PEF, peak expiratory flow; MMEF, maximal mid-expiratory flow; FEF_75_, forced expiratory flow at 75% of FVC; FEF_50_, forced expiratory flow at 50% of FVC; FEF_25_, forced expiratory flow at 25% of FVC.

As shown in Figure 5, no parameters of lung function were statistically significant between the Qiju group and the control group at baseline. The difference in lung function parameters between these two groups became greater, and the trend to provide lung health benefits tended to increase in the Qiju group in the 2nd and 4th weeks. However, there were no statistical differences in all time periods (Figure 5).

## 4 Discussion

In this randomized trial among healthy young adults in Hangzhou, China, we examine lung function benefits of Qiju granules against PM_2.5_ exposure at different time periods occurring at lag intervals of 0, 1, or 0–1 week before health examination, which include large airways function (FVC, FEV_1_, and PEF) and small airways function (MMEF, FEF_75_, FEF_50_, and FEF_25_). Our major findings include the following: (1) PM_2.5_ exposure concentration is associated with reduced lung function parameters, including FEV_1_, PEF, MMEF, FEF_75_, FEF_50_, and FEF_25_ in healthy adults. High-level PM_2.5_ exposure has a stronger correlation with a decrease in lung function. PM_2.5_ may impair small airway function more seriously than large airway function (2). The impact of PM_2.5_ on lung function depends on the time lag (3). Qiju granules may alleviate the decrease in lung function after PM_2.5_ exposure.

Some studies have reported significantly different sensitivity to air pollution between male and female subjects (18–20), and others have found significant differences in the association between lung function and air pollution according to BMI (6, 21). Guo et al. (6) reported that the association between FEV_1_ and air pollution was stronger for the participants with low and normal BMI (BMI < 23 kg/m^2^). Siddique et al. (21) reported that the association between lung function and air pollution was stronger for both low normal BMI and obese participants compared with normal weight participants. Those studies suggest that sex and BMI may have an impact on the association between lung function and air pollution exposure. Therefore, we used linear mixed-effects models, adjusted sex and BMI to evaluate the association between PM_2.5_ exposure concentrations and lung function outcomes within each group at different time periods.

The association between PM_2.5_ exposure concentrations and lung health has been investigated in detail. Numerous epidemiology studies provided evidence that PM_2.5_ exposure damages lung function (6, 22, 23). A longitudinal cohort study indicates that each 10 μg/m^3^ increase in PM_2.5_ exposure concentrations is associated with decreases in FVC and FEV_1_ (6). A cross-sectional study of adults from Shanghai, China has shown that higher PM_2.5_ exposure concentrations are significantly associated with lower FVC, IC, and VC (12). PM_2.5_ is a main pollutant of ambient air pollution. The adverse effect on lung health is mostly attributed to its smaller particle size, larger surface area, physical characteristics of the particles, and stronger activity. In addition, PM_2.5_ may be composed of sulfur dioxide, sulfates, metals, nitrates, acids, and carbon particles with various chemicals adsorbed onto their surfaces (24). A recent study in an animal model demonstrated that PM_2.5_ led to easier deposition into lung tissues compared with other pollutants (25). The deposition of PM_2.5_ increases the expression of inflammatory cytokine by activating inflammatory-associated cells and triggers oxida-tive stress by impairing the immune balance (25). Some studies indicate that PM_2.5_ is a critical factor for overall lung function impairment progression by the regulation of oxidative stress in the lungs and inflammation in mice (26, 27). Hence, anti-oxidation and anti-inflammatory supplementation may protect people from the adverse effects of PM_2.5_ on lung function.

Qiju granules are extracted from traditional Chinese herbs, including *Rhodiola rosea L*. [Crassulaceae], *Portulaca oleracea L*. [Portulacaceae], *Lycium barbarum L*. [Solanaceae], *Chrysanthemum* × *morifolium (Ramat.) Hemsl*. [Asteraceae] and *Siraitia grosvenorii* (Swingle) *C.Jeffrey ex A.M.Lu & Zhi Y.Zhang* [Cucurbitaceae]. *Rhodiola rosea L*. [Crassulaceae] is often used as a part of the regimen to treat lung disorders. The main constituent of *Rhodiola rosea L*. [Crassulaceae] is salidroside, which shows attenuation in inflammation and oxidative stress response (12, 13). *Portulaca oleracea L*. [Portulacaceae] is a nutritious vegetable, which is useful for the prevention of diseases caused by oxidative stress. *Lycium barbarum L*. [Solanaceae] exhibits anti-oxidative and anti-inflammatory effects by modulating mRNA expression in many diseases (14). *Chrysanthemum* × *morifolium (Ramat.) Hemsl*. [Asteraceae] has been widely consumed as tea for centuries. The combinations of *Chrysanthemum* × *morifolium (Ramat.) Hemsl*. [Asteraceae] and *Lycium barbarum L*. [Solanaceae] also possess anti-oxidation and anti-inflammation properties by inhibiting MAPKs and NF-kB pathways (15). *Siraitia grosvenorii* (Swingle) *C.Jeffrey ex A.M.Lu & Zhi Y.Zhang* [Cucurbitaceae] has been used as a natural sweetener and as a traditional medicine for the treatment of pharyngitis and tussis. Its active constituents also show anti-oxidative, hypoglycemic, immunologic, anti-tussive, and sputum-reducing efficacy (16). These traditional Chinese herbs are widely used to treat and prevent lung disorders.

Our study demonstrates that exposure to PM_2.5_ concentration induced lung function impairment. However, the results indicate that only some lung function parameters are consistent with previous studies. In our study, we find an 8.80% decrease in the percentage of estimate of FEV_1_, and a 7.07% decrease in the percentage of estimate of PEF per 10 μg/m^3^ increase in PM_2.5_ exposure concentrations. These results are stronger than those reported in other studies (6, 28). This discrepancy may be attributable to the higher PM_2.5_ concentrations in our study time periods. We also measured small airway function parameters, including MMEF, FEF_75_, FEF_50_, and FEF_25_. Only a few previous studies have examined MMEF, FEF_75_, FEF_50_, and FEF_25_. We suggest that those are important parameters of small airway function that are critical in small airway disease development, such as asthma and chronic obstructive pulmonary disease (COPD) (29). In our study, we demonstrated that PM_2.5_ exposure concentrations generally had a stronger association with MMEF, FEF_75_, FEF_50_, and FEF_25_ than FEV_1_ and PEF. There is a 13.07% decrease in the percentage of the estimate of MMEF, a 13.38% decrease in FEF_75_, a 12.53% decrease in FEF_50_, a 9.67% decrease in FEF_25_ per 10 μg/m^3^ of increase in PM_2.5_ exposure concentration. However, there is only an 8.80% decrease in FEV_1_, a 7.07% decrease in PEF, and no significant decrease in FVC. It seems that our findings are consistent with several previous studies (6, 20, 22), which suggest that PM_2.5_ may have more serious impacts on small airway function than on large airway function.

Previous studies indicated that PM_2.5_ induced a significant delayed effect on population health (6, 23, 30, 31). In our study, we show that most lung function parameters are significantly correlated with weekly average PM_2.5_ exposure concentrations of lag 1 week, although other periods of average PM_2.5_ concentrations (lag 0 and 0–1 week) are not significantly correlated with most parameters of lung function, which seems different when compared with some previous studies. We assume it may be primarily due to the fact that weekly average PM_2.5_ concentrations were applied instead of daily average concentrations in those analyses while lag 1 week average concentrations were much higher than those in other time periods. It suggests that high-level PM_2.5_ exposure may have a stronger correlation with lung function, and the impact of PM_2.5_ on lung health does have time lags, which could be acute or even lifetime.

Our study also demonstrates that Qiju granules protect against PM_2.5_ induced lung function impairment, especially in small airway functions. In the control group, FEV_1_, PEF, MMEF, FEF_75_, FEF_50_, and FEF_25_ were significantly decreased when PM_2.5_ exposure concentrations were increased. However, this did not happen in the Qiju group when PM_2.5_ exposure concentrations were increased, which suggests that Qiju may have a preventive effect on small airway functions against PM_2.5_ exposure-related injury. This reason may be due to the active constituents of Qiju granules being able to attenuate inflammation, oxidative stress, and fibrosis response, protecting people from the adverse effects of PM2.5 on lung function. We also compare the change in parameters of lung function between the Qiju group and the control group when adjusting the PM_2.5_ exposure concentrations. Our data demonstrate that the majority of lung function parameters are slightly higher in the Qiju group than in the control group in the 2nd week and 4th week. Unfortunately, there is no significant difference between the two groups in all lung function parameters during these time periods. This could be attributed to our limited enrollment size (40) along with the brief duration of the study (4 weeks), which might not provide sufficient power to demonstrate any further potential lung function benefits.

## 5 Strengths

This study has several strengths. The first strength of the study is reducing the effects of psychological and other confounding factors on outcomes by double-blinded and placebo control. The second strength of the study is monitoring PM_2.5_ exposure concentrations with personal and portable devices, rather than estimating exposures based on outdoor monitors, are precise real-time personal exposures and variation range of exposures for regression analysis. The third strength of the study is that it is conducted among healthy college students on campus, rather than in an exposure-controlled environment, which is closer to real-world situations where local residents live. The fourth strength of the study is that the participants are healthy college students, who are a vulnerable population to PM_2.5_ exposure (32). In addition, the season of the study period has relatively high PM_2.5_ concentrations, which occur frequently in major cities in China and in other developing countries. It has enabled us to obtain stable and precise estimates. Finally, we analyze numerous lung function parameters, including large and small airway function parameters, and use mixed-effects models, adjusting sex and BMI to evaluate PM_2.5_ exposure in relation to lung functions. It provides more stable and reliable results.

## 6 Limitations

This study also has several limitations. The first limitation is the study sample, comprising only 20 participants per group, which consisted of healthy individuals within a relatively ideal experimental setting. Thus, caution should be exercised when generalizing these findings to the broader population and real-world contexts. The second limitation is that healthy adults may be less susceptible to the adverse effects of PM_2.5_ exposure on lung dysfunction and respiratory disorders compared with vulnerable populations, such as children and seniors. The lung function benefits from Qiju granules await further verification with more susceptible and diverse populational studies. The third limitation is that other air pollutants, such as SO_2_, NO_2_, and Ozone, and meteorological parameters, including temperature and humidity, are not collected and analyzed. Therefore, we cannot distinguish the association between PM_2.5_ and lung dysfunction due to PM_2.5_ specifically or in conjunction with other air pollutants and meteorological factors. Finally, although we adjust sex and BMI for the association between PM_2.5_ and lung function, more confounding factors, such as behavior, diet, and living environment, should be considered in future studies.

## 7 Conclusion

In summary, PM_2.5_ exposure is associated with reduced lung function parameters. Qiju granules significantly alleviate the decrease in lung function after PM_2.5_ exposure, which may be effective in protecting lung health against adverse effects of PM_2.5_ exposure.

## Data availability statement

The original contributions presented in the study are included in the article/Supplementary material, further inquiries can be directed to the corresponding author.

## Ethics statement

The studies involving humans were registered in Chinese Clinical Trial Registry (ChiCTR1900021235) on 13 February 2019 and was approved by the Ethics Committee of the First Affiliated Hospital of Zhejiang Chinese Medical University. The studies were conducted in accordance with the local legislation and institutional requirements. The participants provided their written informed consent to participate in this study.

## Author contributions

RC: Writing – original draft. LuZ: Writing – original draft. WG: Writing – original draft. RL: Writing – original draft. HH: Writing – original draft. LiZ: Writing – original draft. JZ: Writing – original draft. YW: Writing – original draft. PN: Writing – original draft. SX: Writing – original draft. ZW: Writing – review & editing. QS: Writing – review & editing. CL: Writing – review & editing. JY: Writing – review & editing.

## References

[1] Liu C Chen R Sera F Vicedo-Cabrera AM Guo Y Tong S. Ambient particulate air pollution and daily mortality in 652 cities. N Engl J Med. (2019) 381:705–15. 10.1056/NEJMc1913285 31433918 PMC7891185

[2] Pun VC Kazemiparkouhi F Manjourides J Suh HH. Long-Term PM2. 5 exposure and respiratory, cancer, and cardiovascular mortality in older US adults. Am J Epidemiol. (2017) 186:961–9. 10.1093/aje/kwx166 28541385 PMC6915823

[3] Collaborators GBDRF. Global, regional, and national comparative risk assessment of 84 behavioural, environmental and occupational, and metabolic risks or clusters of risks for 195 countries and territories, 1990-2017: a systematic analysis for the Global Burden of Disease Study 2017. Lancet. (2018) 392:1923–94. 10.1016/S0140-6736(18)32225-6 30496105 PMC6227755

[4] Organization WH. Out of 10 People Worldwide Breathe Polluted Air, But More Countries are Taking Action. (2018). Available online at: http://wwwwhoint/news-room/detail/02-05-2018-9-out-of-10-people-worldwide-breathe-polluted-air-but-more-countries-are-taking-action (accessed May 2, 2018).

[5] Chen R Zhao A Chen H Zhao Z Cai J Wang C. Cardiopulmonary benefits of reducing indoor particles of outdoor origin: a randomized, double-blind crossover trial of air purifiers. J Am Coll Cardiol. (2015) 65:2279–87. 10.1016/j.jacc.2015.03.553 26022815 PMC5360574

[6] Guo C Hoek G Chang LY Bo Y Lin C Huang B. Long-term exposure to ambient fine particulate matter (PM2. 5) and lung function in children, adolescents, and young adults: a longitudinal cohort study. Environ Health Perspect. (2019) 127:127008. 10.1289/EHP5220 31873044 PMC6957275

[7] Pacitto A Amato F Moreno T Pandolfi M Fonseca A Mazaheri M. Effect of ventilation strategies and air purifiers on the children's exposure to airborne particles and gaseous pollutants in school gyms. Sci Total Environ. (2020) 712:135673. 10.1016/j.scitotenv.2019.135673 31810696

[8] Ni L Chuang CC Zuo L. Fine particulate matter in acute exacerbation of COPD. Front Physiol. (2015) 6:294. 10.3389/fphys.2015.00294 26557095 PMC4617054

[9] Wang J Xiong X Yang G Zhang Y Liu Y Zhang Y. Chinese herbal medicine qi ju di huang wan for the treatment of essential hypertension: a systematic review of randomized controlled trials. Evid Based Complement Alternat Med. (2013) 2013:262685. 10.1155/2013/262685 23878593 PMC3708442

[10] Chau C. The Chinese traditional diet: a socioecological approach. J Austr Trad Med Soc. (2019) 25:222–4.

[11] Shahrajabian M Sun W Zandi P Cheng Q A. review of chrysanthemum, the eastern queen in traditional Chinese medicine with healing power in modern pharmaceutical sciences. Appl Ecol Environ Res. (2019) 17:13355–69. 10.15666/aeer/1706_1335513369

[12] Hu R Wang MQ Ni SH Wang M Liu LY You HY. Salidroside ameliorates endothelial inflammation and oxidative stress by regulating the AMPK/NF-kappaB/NLRP3 signaling pathway in AGEs-induced HUVECs. Eur J Pharmacol. (2020) 867:172797. 10.1016/j.ejphar.2019.172797 31747547

[13] Pu WL Zhang MY Bai RY Sun LK Li WH Yu YL. Anti-inflammatory effects of *Rhodiola rosea* L.: A review. Biomed Pharmacother. (2020) 121:109552. 10.1016/j.biopha.2019.109552 31715370

[14] Lee YJ Ahn Y Kwon O Lee MY Lee CH Lee S. Dietary wolfberry extract modifies oxidative stress by controlling the expression of inflammatory mRNAs in overweight and hypercholesterolemic subjects: a randomized, double-blind, placebo-controlled trial. J Agric Food Chem. (2017) 65:309–16. 10.1021/acs.jafc.6b04701 28027641

[15] Zhang N He Z He S Jing P. Insights into the importance of dietary chrysanthemum flower (*Chrysanthemum morifolium* cv. Hangju)-wolfberry (*Lycium barbarum* fruit) combination in antioxidant and anti-inflammatory properties. Food Res Int. (2019) 116:810–8. 10.1016/j.foodres.2018.09.015 30717012

[16] Gong X Chen N Ren K Jia J Wei K Zhang L. The fruits of *Siraitia grosvenorii:* a review of a Chinese food-medicine. Front Pharmacol. (2019) 10:1400. 10.3389/fphar.2019.01400 31849659 PMC6903776

[17] Xu S Yang J Liu C Chen R Wang Z Hong H. Preventive effect of Qiju decoction on acute lung injury caused by PM2. 5 in mice. Zhejiang Clin Med J. (2020) 22:617–9.

[18] Gauderman WJ Vora H McConnell R Berhane K Gilliland F Thomas D. Effect of exposure to traffic on lung development from 10 to 18 years of age: a cohort study. Lancet. (2007) 369:571–7. 10.1016/S0140-6736(07)60037-3 17307103

[19] Gauderman WJ Urman R Avol E Berhane K McConnell R Rappaport E. Association of improved air quality with lung development in children. N Engl J Med. (2015) 372:905–13. 10.1056/NEJMoa1414123 25738666 PMC4430551

[20] Oftedal B Brunekreef B Nystad W Madsen C Walker SE Nafstad P. Residential outdoor air pollution and lung function in schoolchildren. Epidemiology. (2008) 19:129–37. 10.1097/EDE.0b013e31815c0827 18091005

[21] Siddique S Banerjee M Ray MR Lahiri T. Air pollution and its impact on lung function of children in Delhi, the capital city of India. Water, Air, & Soil Pollution. (2010) 212:89–100. 10.1007/s11270-010-0324-1

[22] Hwang BF Chen YH Lin YT Wu XT Leo Lee Y. Relationship between exposure to fine particulates and ozone and reduced lung function in children. Environ Res. (2015) 137:382–90. 10.1016/j.envres.2015.01.009 25614339

[23] Xu D Zhang Y Zhou L Li T. Acute effects of PM2. 5 on lung function parameters in schoolchildren in Nanjing, China: a panel study. Environ Sci Pollut Res Int. (2018) 25:14989–95. 10.1007/s11356-018-1693-z 29550979

[24] Hu Y Zhou Q Liu T Liu Z. Coixol suppresses NF-kappaB, MAPK pathways and NLRP3 inflammasome activation in lipopolysaccharide-induced RAW 264.7 Cells. Molecules. (2020) 25:894. 10.3390/molecules25040894 32085388 PMC7070437

[25] Li Q Liu H Alattar M Jiang S Han J Ma Y. The preferential accumulation of heavy metals in different tissues following frequent respiratory exposure to PM2. 5 in rats Sci Rep. (2015) 5:16936. 10.1038/srep16936 26582271 PMC4652264

[26] Jiang S Bo L Du X Liu J Zeng X He G. CARD9-mediated ambient PM2. 5-induced pulmonary injury is associated with Th17 cell. Toxicol Lett. (2017) 273:36–43. 10.1016/j.toxlet.2017.03.015 28315386

[27] Gu LZ Sun H Chen JH. Histone deacetylases 3 deletion restrains PM2. 5-induced mice lung injury by regulating NF-kappaB and TGF-beta/Smad2/3 signaling pathways. Biomed Pharmacother. (2017) 85:756–62. 10.1016/j.biopha.2016.11.094 27919737

[28] Gehring U Gruzieva O Agius RM Beelen R Custovic A Cyrys J. Air pollution exposure and lung function in children: the ESCAPE project. Environ Health Perspect. (2013) 121:1357–64. 10.1289/ehp.1306770 24076757 PMC3855518

[29] Gilliland FD Berhane K McConnell R Gauderman WJ Vora H Rappaport EB. Maternal smoking during pregnancy, environmental tobacco smoke exposure and childhood lung function. Thorax. (2000) 55:271–6. 10.1136/thorax.55.4.271 10722765 PMC1745733

[30] Hsu SC Chang JH Lee CL Huang WC Hsu YP Liu CT. Differential time-lag effects of ambient PM2. 5 and PM25-bound PAHs on asthma emergency department visits. Environ Sci Pollut Res Int. (2020) 27:43117–24. 10.1007/s11356-020-10243-y 32729038

[31] Chu H Xin J Yuan Q Zhang X Pan W Zeng X. Evaluation of vulnerable PM2. 5-exposure individuals: a repeated-measure study in an elderly population. Environ Sci Pollut Res Int. (2018) 25:11833–40. 10.1007/s11356-018-1412-9 29446019

[32] Sly PD Flack F. Susceptibility of children to environmental pollutants. Ann N Y Acad Sci. (2008) 1140:163–83. 10.1196/annals.1454.017 18991915

