# Supplementary material for: Lung function benefits of traditional Chinese medicine Qiju granules against fine particulate air pollution exposure: a randomized controlled trial
Source: Front Med (Lausanne). 2024 Apr 29;11:1370657. doi: 10.3389/fmed.2024.1370657 (PMC11089203; doi:10.3389/fmed.2024.1370657)
Supplement: Supplementary file 2 [file Table_1.DOCX]

Supplemental Table 1. Parameters of airbeam instrument calibration

| **Portable Monitor** | **B_max_** | **K_d_** | **R^2^** |
| --- | --- | --- | --- |
| **Airbeam1** | 225.7 | 420.7 | 0.96 |
| **Airbeam2** | 178.6 | 283.7 | 0.90 |
| **Airbeam3** | 239.9 | 434.8 | 0.98 |
| **Airbeam4** | 218.1 | 374.3 | 0.95 |
| **Airbeam5** | 246.8 | 402.0 | 0.95 |
| **Airbeam6** | 249.1 | 378.7 | 0.96 |
| **Airbeam7** | 221.8 | 326.2 | 0.90 |
| **Airbeam8** | 241.6 | 447.5 | 0.97 |
| **Airbeam9** | 229.2 | 368.1 | 0.95 |
| **Airbeam10** | 166.7 | 236.7 | 0.96 |
| **Airbeam11** | 162.4 | 239.2 | 0.97 |
| **Airbeam12** | 191.6 | 328.7 | 0.95 |
| **Airbeam13** | 168.7 | 259.0 | 0.97 |
| **Airbeam14** | 188.6 | 288.4 | 0.91 |
| **Airbeam15** | 191.1 | 308.2 | 0.94 |
| **Airbeam16** | 163.5 | 257.0 | 0.95 |
