# Supplementary material for: Lung function benefits of traditional Chinese medicine Qiju granules against fine particulate air pollution exposure: a randomized controlled trial
Source: Front Med (Lausanne). 2024 Apr 29;11:1370657. doi: 10.3389/fmed.2024.1370657 (PMC11089203; doi:10.3389/fmed.2024.1370657)
Supplement: Supplementary file 3 [file Table_2.DOCX]

Supplemental Table 2. Biomarkers of blood routine, hepatic function, renal function, urine routine at the beginning and end of the trial

| **Biomarker** | **Baseline (N=20)^*^** | | **After intervention (N=20)^*^** | |
| --- | --- | --- | --- | --- |
|  | **Treatment Group** | **Control Group** | **Treatment Group** | **Control Group** |
| **Blood routine** |  |  |  |  |
| **WBC (10^9/L, Mean ± SD)** | 5.87 ± 1.42 | 5.98 ± 1.20 | 5.84 ± 1.35 | 5.10 ± 1.45 |
| **RBC (10^12/L, Mean ± SD)** | 4.78 ± 0.60 | 4.93 ± 0.72 | 4.70 ± 0.53 | 4.51 ± 0.42 |
| **HGB (g/L, Mean ± SD)** | 145.16 ± 16.61 | 144.45 ± 13.06 | 143.05 ± 15.23 | 137.80 ± 12.51 |
| **PLT (10^9/L, Mean ± SD)** | 242.05 ± 41.35 | 251.95 ± 62.38 | 231.84 ± 2.131 | 216.80 ± 57.58 |
| **NEUT# (10^9/L, Mean ± SD)** | 3.88 ± 1.14 | 3.96 ± 1.42 | 3.41 ± 1.33 | 3.05 ± 0.51 |
| **LYMPH# (10^9/L, Mean ± SD)** | 1.58 ± 0 .38 | 1.78 ± 0.39 | 1.80 ± 0.32 | 1.79 ± 0.52 |
| **MONO# (10^9/L, Mean ± SD)** | 0.35 ± 0.23 | 0.31 ± 0.14 | 0.33 ± 0.08 | 0.32 ± 0.10 |
| **EO# (10^9/L, Mean ± SD)** | 0.08 ± 0.05 | 0.09 ± 0.08 | 0.10 ± 0.06 | 0.12 ± 0.11 |
| **BASO# (10^9/L, Mean ± SD)** | 0.02 ± 0.01 | 0.01 ± 0.01 | 0.04 ± 0.06 | 0.03 ± 0.02 |
| **Hepatic function** |  |  |  |  |
| **TBL (μmol/L, Mean ± SD)** | 13.96 ± 4.25 | 12.16 ± 3.62 | 9.82 ± 4.81 | 10.83 ± 4.05 |
| **TP (g/L, Mean ± SD)** | 70.91 ± 3.21 | 71.70 ± 2.64 | 72.13 ± 2.94 | 70.53 ± 2.64 |
| **ALT (U/L, Mean ± SD)** | 18.20 ± 13.20 | 17.30 ± 10.99 | 19.65 ± 13.82 | 16.40 ± 10.40 |
| **AST (U/L, Mean ± SD)** | 17.80 ± 3.25 | 18.05 ± 4.52 | 19.70 ± 4.85 | 18.05 ± 3.59 |
| **ALP (U/L, Mean ± SD)** | 77.25 ± 17.35 | 80.05 ± 19.61 | 76.95 ± 14.17 | 80.50 ± 19.44 |
| **Renal function** |  |  |  |  |
| **UA (μmol/L, Mean ± SD)** | 324.1 ± 65.82 | 338.85 ± 87.80 | 313.50 ± 79.00 | 326.00 ± 81.01 |
| **CR (μmol/L, Mean ± SD)** | 61.85 ± 10.98 | 63.80 ± 13.15 | 64.20 ± 10.50 | 63.75 ± 13.09 |
| **urea (mmol/L, Mean ± SD)** | 4.55 ± 0.85 | 4.57 ± 1.47 | 4.87 ± 1.06 | 4.34 ± 1.06 |
| **Urine routine** |  |  |  |  |
| **PH (Mean ± SD)** | 6.13 ± 0.36 | 6.16 ± 0.37 | 6.11 ± 0.42 | 6.17 ± 0.54 |
| **SG (Mean ± SD)** | 1.02 ± 0.006 | 1.02 ± 0.005 | 1.02 ± 0.005 | 1.02 ± 0.005 |

Abbreviations: WBC, white blood cells; RBC, red blood cells; HGB, hemoglobin; PLT, platelets; NEUT#, absolute neutrophil count; LYMPH#, absolute lymphocyte count; MONO#, absolute monocyte count; EO#, absolute eosnophils count; BASO#, absolute basophils count; TBL, total bilirubin; TP, total protein; ALT, alanine aminotransferase; AST, aspartate aminotransferase; ALP, alkaline phosphatase; UA, uric acid; CR, creatinine; SG, specific gravity.

* All biomarkers were not statistically different between the two groups at baseline and end of trial.
