# Supplementary material for: Lung function benefits of traditional Chinese medicine Qiju granules against fine particulate air pollution exposure: a randomized controlled trial
Source: Front Med (Lausanne). 2024 Apr 29;11:1370657. doi: 10.3389/fmed.2024.1370657 (PMC11089203; doi:10.3389/fmed.2024.1370657)
Supplement: Supplementary file 4 [file Table_3.DOCX]

Supplemental Table 3. PM_2.5_ concentrations in different periods before health examination (μg/m^3^)

| **Lag Time*** | **Mean ± SD** | **Min** | **Median** | **Max** | **IQR** |
| --- | --- | --- | --- | --- | --- |
| **0 Week** | 57.58 ± 6.26 | 42.08 | 58.66 | 72.67 | 8.32 |
| **1 Week** | 80.12 ± 6.53 | 66.01 | 79.47 | 99.49 | 6.48 |
| **0-1 Week** | 68.85 ± 4.63 | 60.08 | 68.68 | 79.69 | 5.70 |

Abbreviations: SD, standard deviation; IQR, interquartile range; Min, minimum; Max, maximum.

*lag 0 week mean 7 days before examination day; lag 1 week means the 8^th^ day to the 14^th^ day before examination; lag 0-1 week means 14 days before examination day.
