# Supplementary figures and images for: Lung function benefits of traditional Chinese medicine Qiju granules against fine particulate air pollution exposure: a randomized controlled trial
Source: Front Med (Lausanne). 2024 Apr 29;11:1370657. doi: 10.3389/fmed.2024.1370657 (PMC11089203; doi:10.3389/fmed.2024.1370657)

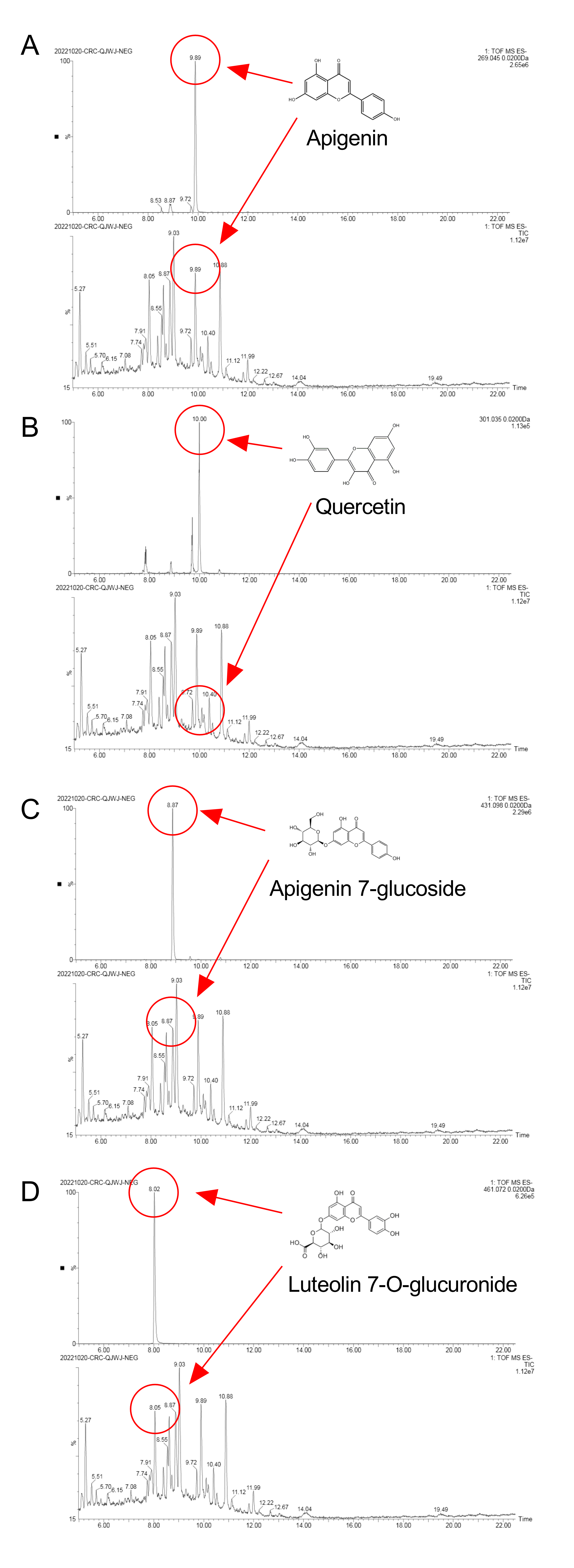

Supplement: Supplementary file 5 [file Image_1.TIF]

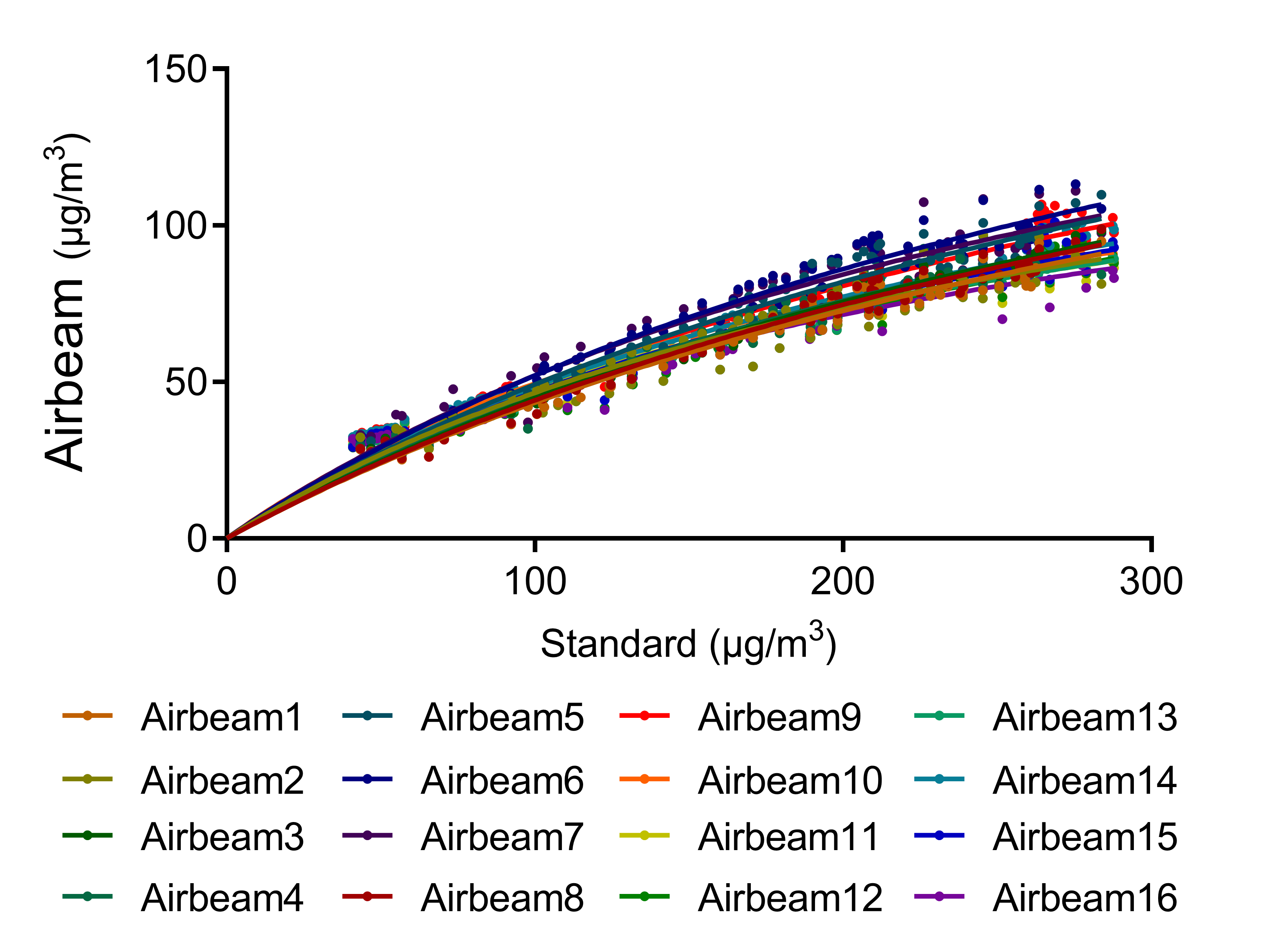

Supplement: Supplementary file 6 [file Image_2.TIF]
